# Ex Vivo Generated Red Cells as Transfusion Products

**DOI:** 10.1155/2012/615412

**Published:** 2012-04-18

**Authors:** Anna Rita Migliaccio, Giuliano Grazzini, Christopher D. Hillyer

**Affiliations:** ^1^Tisch Cancer Institute, Mount Sinai School of Medicine, New York, NY 10029, USA; ^2^National Blood Centre, 00161 Rome, Italy; ^3^New York Blood Center and Weill Cornell Medical College, New York, NY 10065, USA

This issue of the Stem Cell International journal contains papers from many of the leading scientists in the emerging field: ex vivo expansion of hematopoietic progenitor cells into erythrocytes for transfusion.

Blood transfusion, the first form of successful cell therapy and, at least to some, “transplantation”, was inspired by the discovery of the circulation by Richard Harvey in the 1600s [1] and begun in earnest later in that century. The development of this clinical practice into the safe and routine therapy we all know today has been both an exciting scientific adventure and the foundation for a number of other scientific disciplines. More specifically, the *immunology* of transfusion and transplantation began with the discovery of the heterogeneity of human blood group antigens by Dr. Karl Landsteiner in 1901 (recognized with a Nobel Prize in 1930). The discovery of clinically relevant infectious diseases transmitted by transfusion played an important role in the development and advancement of *virology*. The inheritance of certain form of anemias was discovered during blood transfusion practice and led to development of the *genetics* of human red cell disorders. In the 1940–1950s, the establishment of blood banks followed by the development of rigorous donation criteria and standardization of blood manufacturing processes has made transfusion safe and widely available and has provided a paradigm for the development of emerging therapies using ex vivo expansion and differentiation of many cell types. An example of one such therapy is represented by the tumor immunotherapy described by Lapteva and Vera.

The blood supply of industrialized countries is adequate overall. Nearly one hundred million donations are made every year worldwide (http://www.who.int/mediacentre/factsheets/fs279/en/index.html). The availability of blood and blood products in these nations has permitted the development and implementation of numerous life-saving surgical procedures (open heart surgery, organ transplantation, damage control resuscitation for trauma, and others) and cancer treatments which were not even imaginable without assurance that blood for transfusion would be readily available and safe. However, blood is not an unlimited resource and its potential need as the world rapidly develops requires a significant increase in blood donation. By some estimates (CDH), given the world's population and given the per capita transfusion of Canada as a utilization benchmark, nearly 250 million whole blood donations would be needed. Furthermore, and despite its high level of safety, human donated, unit-by-unit-derived blood donation/transfusion (i.e., without batched blood manufacturing into an aliquoted and homogenized pharmaceutical product), still leads to morbidity and mortality of its own accord and has significant variation from product to product based on the nature of the collection, manufacturing and storage processes, and the antigenic variation of any given donor, amongst others. Finally, it is not known what effect the aging of the world's population will have both on per capita utilization and on the ability of the smaller, younger populations to donate [2]. These issues, and the nearly 20-year-old search for alternative products to meet the transfusion need are discussed in the paper by Whitsett et al.

Scientific research is inspired by the prospect of a clinical goal. In recent years, a revolution in stem cell biology has occurred that has far reaching implications, specifically, the discovery that it is possible to generate a potentially unlimited supply of stem cells by epigenetic/genetic treatments of somatic cells (T cells, fibroblasts, others) from any individual (see Pourcher et al., Hyroyama et al., and Chang et al.). In addition, techniques have been discovered to reprogram any cell into another cell type avoiding the induction of pluripotency. These techniques are fascinating though there are numerous scientific, safety, and scaling-up issues to be resolved before cells which have been genetically “altered” in the laboratory may be considered ready for widespread clinical use. As red blood cells do not have a nucleus, it is possible that they will be accepted as genetically safe. Indeed, it is this notion that supports that red blood cells from Hematopoietic stem/progenitor cell expansion or redifferentiation may represent the first therapeutic product to be generated by genomic reprogramming technology.

Reprogramming technology is still under development. Therefore, red blood cells expanded *ex vivo* from primary stem cell sources currently discarded (buffy coats produced during the blood manufacturing processes and low-volume umbilical cord blood) are being considered for first-in-man studies. Tirelli et al. identify the cell populations present in adult blood which are responsible for massive production of red blood cells *ex vivo*. The first-in-man proof-of-principle study for the use of *in vitro* expanded red blood cells for transfusion was reported on September 1st 2011, by Luc Douay and colleagues [3], who have also coauthored Pourcher et al. This paper reported that red blood cells generated *in vitro* from mobilized CD34^pos^ cells collected by apheresis have normal survival (determined by ^51^Cr labeling) when transfused into an autologous recipient [3]. This first-in-man autologous transfusion described also what would be the most likely safety data necessary for a larger clinical study with such products [*in vitro* characterization (blood group antigen expression profiling, deformability, hemoglobin content and O_2_ dissociation curves) and *in vivo* functional studies in animal models (survival and morphology); http://www.clinicaltrials.gov/ct2/show/NCT00929266]. *In vivo* functional studies of human red blood cells in animal models will likely allow more complete characterization in many ways [4]. Ghinassi et al. describe an improved animal model which allows *in vivo* imaging and cell fate determination of human erythroid cells by labeling the cells before transfusion with a fluorescent reporter gene by retroviral technology.

Although red blood cells do not have nuclei, their immediate precursors the erythroblasts do. The terminal maturation of erythroblasts into functional red cells requires a complex remodeling process which ends with extrusion of the nucleus and the formation of an enucleated red blood cell [5]. These late stages of maturation are intrinsically controlled by epigenetic/genetic expression programs of the erythroblast itself. Cell reprogramming methodologies may (and at present appear to) disrupt these programs, leading to inefficient enucleation. Keerthivasan et al. discuss novel insights into the critical mechanisms of terminal maturation of a red blood cell and strategies to improve the efficiency of these processes.

As represented by all the information, data, and in fact vision contained in this issue, we are clearly at the beginning of a rapidly expanding field. The papers herein provide a broad and comprehensive overview of the most relevant areas of research which have been pursued and are needed to advance the field. Still, as state of the art as this issue is presently, the field is moving so rapidly that one may predict that new knowledge will rapidly follow.

*Anna Rita Migliaccio* *Anna Rita Migliaccio* *Giuliano Grazzini* *Giuliano Grazzini* *Christopher D. Hillyer* *Christopher D. Hillyer*
